# Medical Items

**Published:** 1897-10

**Authors:** 


### MEDICAL ITEMS.

A Southern editor makes this announcement about his journal: "A first-class newspaper, entered as second-class matter in a third-class post-office."—*Ex.*

Da Costa says that sulphur and cream of tartar, equal parts, form an excellent laxative for hemorrhoidal conditions during pregnancy, producing soft and semi-liquid stools.

From 12 to 20 grains of ammonio-ferric-alum dissolved in eight ounces of water, and gargled at frequent intervals, will relieve ordinary cases of "sore throat" within a few hours.—*Med. News.*

Our esteemed neighbor, the *Alabama Medical and Surgical Age,* has removed from Anniston to Birmingham. A wise move, no doubt. The *Age* should now remove its insert advertising pages.

Dr. Chas. M. Blackford has resigned the place of Demonstrator of Pathology and Bacteriology in the Atlanta Medical College, to accept the Professorship of Pathology in the Medical Department of the University of Georgia at Augusta.

Edward A. Cason, M.D., died of typhoid fever September 16th, aged twenty-seven. Dr. Cason graduated from Bellevue Hospital Medical College in 1891. He moved to LaGrange in 1895 and had built up a large and lucrative practice. He leaves a widow and one daughter, besides parents, sister and host of friends to mourn his early death.

*The Journal* desires to thank the members of the State Medical Examining Board for their compliment in choosing it as their official journal. It is needless to remind our readers of our position relative to the State Board and its work. Our back-numbers contain our record in this regard. We now have a good Board, and we do not doubt that individually and collectively they will discharge their duties without fear or favor.

---

We invite the attention of our readers to the advertisment of Drs. Ekin and Cooper's sanatorium, to be found in our advertising pages. This institution is now completed and ready for patients. It has been especially constructed to meet all the requirements of a modern surgical sanatorium, and is completely furnished with every appliance and apparatus necessary for aseptic work. In the hands of Drs. Elkin and Cooper we are sure the institution will meet with the success which it fully deserves.

---

According to the *American Medical Journal* there are now in the United States and Canada 275 medical periodicals. Ten are Canadian. One hundred and thirty-five are devoted to medicine and surgery; twenty-two to pharmacy and therapeutics; ten to the eye, ear, nose and throat; six to the nervous system; five to gynecology and obstetrics; three to pediatrics, and two to genito-urinary and rectal diseases. There are twenty-three homeopathic journals and eight eclectic. Thirty-one are classed as miscellaneous.

---

A Mr. Miller, of Asheville, wishes to recover an amputated leg. He says that five years ago the leg was afflicted with scrofula, and it was cut off by Dr. J. A. Burroughs. Permission was given by plaintiff to the physician to retain the leg for a time for professional study, the case being a novel and interesting one, but it was, so the plaintiff alleges, to be returned after a reasonable time, so that, as a faithful and important member of his body up to the time of amputation, it might be given honorable burial.

The plaintiff further alleges that a demand for his leg has been refused and that he has been denied the comfort of having it decently interred. He therefore wants the leg or $500.

---

Following are some of the nuggets of wisdom from the June examination papers of the Pennsylvania Board of Medical Examiners: One man said that the "uriniferous tubules secreted the seminal fluid." Another said that the "function of the optic nerve is to contract the pupil and move the eyeball." Another said that in "cerebral hemorrhage the patient may vomit the cerebro-spinal fluid." Another said that in case of a rigid os he "would decapitate or perform craniotomy, or would put on forceps and deliver at once." Another described a new method of doing a version; he "would put his finger in the child's mouth and bring the chin under the os pubis and hold his hand over the mouth to prevent the liquor amnia from choking it." And yet all these men had diplomas!

---

Dr. Bedford Brown, the noted surgeon, died at his residence in Alexandria, Va., September 12, having failed to rally from the effects of a surgical operation performed recently. Dr. Brown was born in North Carolina seventy-four years ago, and was a son of the late Bedford Brown, who represented that State in the United States Senate. He practiced his profession in his native State, and when war was declared in '61 he entered the Confederate service and won honors as an army surgeon. He was Medical Director of the Confederate Army during the Canby campaign in West Virginia. At the close of hostilities between the States Dr. Brown settled in Alexandria, where he has since practiced his profession. He always stood high in medical councils and was looked upon as an authority by his professional associates. He was particularly prominent in the Southern Surgical Association and the Virginia Medical Society.

The American Pediatric Society is making a Collective Investigation of Infantile Scurvy as occurring in North America, and earnestly requests the coöperation of physicians, through their sending of reports of cases, whether these have already been published or not. No case will be used in such a way as to interfere with its subsequent publication by the observer. Blanks containing questions to be filled out will be furnished on application to any one of the committee. A final printed report of the investigation will be sent to those furnishing cases. J. P. Crozer Griffith, M.D., Chairman, 123 S. 18th St., Phila.; William D. Booker, M.D., 853 Park Ave., Baltimore; Charles G. Jennings, M.D., 457 Jefferson Ave., Detroit; Augustus Caille, M.D., 753 Madison Ave., New York City; J. Lovett Morse, M.D., 317 Marlboro St., Boston, Committee.

---

The sixty-fifth annual meeting of the British Medical Association was held in Montreal, Canada, August 31st to September 4th. It was well attended by several hundred of the most prominent medical men of England and the provinces and a number from other countries. Dr. T. G. Roddick, of Montreal, presided. The most notable addresses were delivered by Professor Charles Richet (official representative of the French government), upon "The Work of Pasteur and the Modern Conception of Medicine"; Professor Mitchell Banks, of Liverpool, upon "The Surgeon of Old in War"; Professor William Osler, of Baltimore, upon "British Medicine in Greater Britain"; Professor Herman Biggs, of New York, delivered the Address in Public Medicine, giving a detailed description of the bacteriological work in the Health Department of New York City. Among those present were Lord Lister, Mr. Christopher Heath, Professor Michael Foster, Dr. Henry Barnes, Dr. R. Saundby, etc. The meeting was a most notable gathering of medical men, and from a scientific point of view one of the most successful in the history of the Association.

The twelfth International Medical Congress, which closed at Moscow, Russia, on August 26, was by all odds the most important meeting of medical men that has occurred this year, and perhaps the most important ever held. The character of the addresses stands out in bold contrast with most of those read at Montreal. Of course there was much chaff, even there, but if we can fairly judge from the reports of our foreign exchanges, it bore no such large proportion to the total. New and far-reaching principles were enunciated, experimental results were recited, unsuspected but important relations were revealed, and inductions of pre-eminent value were attempted. Even those addresses that dealt with hackneyed subjects, judging from those already published, had a freshness of logic thrown into the facts that made them temporarily interesting. At the opening of the meeting by his imperial highness the Grand-Duke Sergei Alexandrovitch, the Grand Theatre, one of the most capacious halls in the world, was crowded. It was announced by the Secretary-General at one of the sessions that over 7,300 medical men were there and registered during the week of the convention. The majority were Russians, but Austria sent 800, Germany 800, France 400, England 300, and the United States 120. The heat during most of the week kept many away from the meetings of the sections, and tended to unfit those who did attend from duly appreciating what they heard. The thermometer stood steadily above 88 degrees F. in the shade. The English-speaking delegates, who did not understand Slav, French, or German found it difficult to read the official notices, none of which was in English. There was a contest between the French and the Spanish as to which should get the next Congress, but it was decided against Madrid and in favor of Paris. The first International Congress was held in Paris in 1867, during the first Paris Exhibition, and the thirteenth will be held there during the great exhibition of 1900. Professor Lannelongue, of Paris, is the new president, and Professor Chaufford, the Honorary Secretary-General.—*Amer. Medico-Surg. Bulletin.*

